# Early Progression in Non-Small Cell Lung Cancer (NSCLC) with High PD-L1 Treated with Pembrolizumab in First-Line Setting: A Prognostic Scoring System Based on Clinical Features

**DOI:** 10.3390/cancers13122935

**Published:** 2021-06-11

**Authors:** Antonio Passaro, Silvia Novello, Diana Giannarelli, Emilio Bria, Domenico Galetta, Alain Gelibter, Maria Lucia Reale, Simona Carnio, Emanuele Vita, Alessio Stefani, Pamela Pizzutilo, Valeria Stati, Ilaria Attili, Filippo de Marinis

**Affiliations:** 1 Division of Thoracic Oncology, European Institute of Oncology IRCCS, 20141 Milano, Italy; valeria.stati@ieo.it (V.S.); ilaria.attili@ieo.it (I.A.); filippo.demarinis@ieo.it (F.d.M.); 2 Department of Oncology, Università Degli Studi Di Torino–AOU San Luigi Gonzaga, 10043 Orbassano, Italy; silvia.novello@unito.it (S.N.); realemarialucia@gmail.com (M.L.R.); simona.carnio@libero.it (S.C.); 3 Istituto Nazionale Tumori Regina Elena, IRCCS, 00144 Roma, Italy; diana.giannarelli@ifo.gov.it; 4 Comprehensive Cancer Center, Fondazione Policlinico Universitario Agostino Gemelli IRCCS, Università Cattolica del Sacro Cuore, 00168 Roma, Italy; emilio.bria@policlinicogemelli.it (E.B.); dr.emanuele.vita@gmail.com (E.V.); ale.stef92@gmail.com (A.S.); 5 Medical Thoracic Unit, IRCCS Oncologico Giovanni Paolo II, 70124 Bari, Italy; galetta@oncologico.bari.it (D.G.); pamela.pizzutilo@gmail.com (P.P.); 6 Medical Oncology Unit “B”, Policlinico Umberto I, Sapienza University, 00161 Roma, Italy; alain.gelibter@uniroma1.it

**Keywords:** immunotherapy, pleural metastasis, corticosteroid, prognosis, detrimental effect, NSCLC

## Abstract

**Simple Summary:**

Immune checkpoint inhibitors demonstrated a survival advantage in the first-line setting in patients with non-oncogene addicted non-small cell lung cancer (NSCLC) and PD-L1 expression ≥50%. However, some patients have detrimental effects from this treatment, but no consistent data are available to predict the outcomes and prognosis. Our work aims to identify clinical features related to poor survival in patients treated with first-line immunotherapy to help clinicians assist in better patient treatment selections with an easily assessable tool.

**Abstract:**

Background: Pembrolizumab is approved in monotherapy for the first-line (1L) of advanced or metastatic NSCLC patients with high PD-L1 (≥50%). Despite a proportion of patients achieve long-term survival, about one-third of patients experience detrimental survival outcomes, including early death, hyperprogression, and fast progression. The impact of clinical factors on early progression (EP) development has not been widely explored. Methods: We designed a retrospective, multicenter study involving five Italian centers, in patients with metastatic NSCLC with PD-L1 ≥ 50%, treated with Pembrolizumab in a 1L setting. EP was defined as a progressive disease within three months from pembrolizumab initiation. Baseline clinical factors of patients with and without EP were collected and analyzed. Logistic regression was performed to identify clinical factors associated with EP and an EP prognostic score was developed based on the logistic model. Results: Overall, 321 out of 336 NSCLC patients treated with 1L pembrolizumab provided all the data for the analysis. EP occurred in 137 (42.7%) patients; the median PFS was 3.8 months (95% CI: 2.9–4.7), and median OS was not reached in the entire study population. Sex, Eastern Cooperative Oncology Group (ECOG) performance status (PS), steroids, metastatic sites ≥2, and the presence of liver/pleural metastasis were confirmed as independent factors for EP by multivariate analysis. By combining these factors, we developed an EP prognostic score ranging from 0–13, with three-risk group stratification: 0–2 (good prognosis), 3–6 (intermediate prognosis), and 7–13 (poor prognosis). The area under the curve (AUC) of the model was 0.76 (95% CI: 0.70–0.81). Conclusions: We identified six clinical factors independently associated with EP. We developed a prognostic score model for EP-risk to potentially improve clinical practice and patient selection for 1L pembrolizumab in NSCLC with high PD-L1, in the real-world clinical setting.

## 1. Introduction

The introduction of immune checkpoint inhibitors (ICIs) dramatically improves the survival outcomes in NSCLC patients. Pembrolizumab monotherapy is demonstrated to significantly prolong overall survival (OS) compared to platinum-doublet chemotherapy as a first-line treatment in non-small cell lung cancer (NSCLC) patients with advanced or metastatic disease whose tumors have high PD-L1 immunohistochemistry expression (≥50%) in the absence of *EGFR* or *ALK* gene alterations [1]. Subsequently, it was established as a standard first-line treatment in high PD-L1 NSCLC without actionable gene alterations [2,3]. However, despite about one-third of patients achieve long-term survival, a relevant proportion of patients receiving monotherapy with ICIs experience detrimental effects [4]. Different patterns of poor outcomes have been described, including early death (ED), namely increased number of deaths within the first twelve weeks, hyperprogression (HPD), defined as an increase in the tumor growth rate as compared to radiological imaging obtained before the ICI start, and fast progression (FP), defined as progressive disease radiologically confirmed within six weeks from ICI initiation [5,6,7]. Early deaths occur in up to 20% of patients in the pretreated setting, HPD in 14%, and FP in 8% of evaluable cases. In a recent retrospective study, the ED rate was 31.4% across treatment lines [6,8]. 

Such detrimental outcomes are not reported in patients receiving chemo-immunotherapy combinations, which also demonstrated efficacy compared to chemotherapy alone as a first-line treatment of NSCLC, regardless of PD-L1 expression [9,10,11]. Very recently, real-world data have been presented regarding the comparable survival outcomes among ICI monotherapy and chemo-immunotherapy combinations in the first-line setting [12]. Hence, identifying clinical and/or molecular biomarkers that might predict patients’ prognosis and treatment outcomes is urgently needed to potentially select patients to treat with ICI monotherapy rather than with chemo-immunotherapy combinations. 

In real-world clinical practice, radiological evaluation is not commonly performed within the first six weeks. Additionally, it is unusual to have pre-treatment radiological evaluations in the first-line setting to assess tumor growth before and after ICI treatment. Therefore, nor FP neither HPD can be adequately detected in clinical practice. 

Based on these considerations, we performed a retrospective study to identify clinical factors and develop a prognostic score model predicting the probability to develop early progression (EP), defined as progression of the disease within three months from pembrolizumab initiation. The objective, as the basis of this line of investigation, is to select patients who might most benefit from combination treatments compared to ICI monotherapy in the first-line setting, or in which anticipated radiologic assessment could be recommended to detect early detrimental effects and define alternative treatment approaches.

## 2. Materials and Methods

### 2.1. Patients

We conducted a multicenter retrospective study in five Italian centers. According to local immunohistochemistry testing, the study included patients diagnosed with metastatic NSCLC expressing high PD-L1 levels (≥50%) who received pembrolizumab monotherapy as a first-line approach between 2017 and 2019. Patients’ clinical records were collected, including demographics, baseline clinical features, tumor, and treatment-related data. Only patients with adequate follow-up information, including disease status or death at database lock, and complete clinical records were considered for study analysis.

All the study procedures were carried out by the general authorization to process personal data for scientific research purposes from “The Italian Data Protection Authority” (http://www.garanteprivacy.it/web/guest/home/docweb/-/docwebdisplay/export/2485392, accessed on 15 April 2021). All information regarding subjects was managed using anonymous numerical codes and handled in compliance with the Declaration of Helsinki. According to the aforementioned national guidelines, the study did not require an Ethical Committee approval since it did not affect the clinical management of the involved patients. Informed consent was obtained from all subjects involved in the study.

### 2.2. Endpoints

The primary endpoint was to identify the rate of patients experiencing EP, defined as the occurrence of progressive disease (PD) within three months of pembrolizumab start. Secondary endpoints were overall survival (OS), progression-free survival (PFS) in the real-world population treated with the first-line pembrolizumab. Specific outcomes related to EP were also addressed to investigate EP-related clinical features and develop a prognostic score model for EP risk.

### 2.3. Statistical Analysis

Variables were presented using the median value for continuous variables and percentages (numbers) for categorical variables.

EP was defined as PD (per investigator) within three months (PFS < 3 months) from the date of first-line pembrolizumab start.

OS was defined as the time between pembrolizumab initiation and death from any cause. PFS was defined as the time between pembrolizumab initiation and progression or death.

Median PFS and OS were estimated by using Kaplan–Meier methods. Median follow-up was calculated with the reverse Kaplan–Meier method. The Cox regression model was used for univariate and multivariate analysis on survival outcomes, and data were presented as odds ratios (OR) or hazard ratios (HR) and their 95% confidence interval (CI), as appropriate.

High tumor burden was defined as the presence of either baseline number of metastatic sites >5, or baseline sum of the target lesions’ longest diameters >76 mm, or bulky disease [13,14].

Logistic regression was performed to identify clinical factors associated with EP. An EP prognostic score was developed based on regression β coefficients. 

Statistical significance level was set at *p* < 0.05 for all tests. All statistical analyses were performed with IBM-SPSS version 21.0. 

## 3. Results

### 3.1. Patient Population

Overall, 336 patients were included in the analysis (Table 1). The median age was 69 years (range 36–86), male to female ratio was 2:1. Patients were predominantly ever smokers (84.8%) and with non-squamous histology (78.6%). Eastern Cooperative Oncology Group (ECOG) performance status (PS) ≥2 patients represented 13.1% of the included population. About three-quarters (73%) of patients presented with at least one major comorbidity, and most of the included population (84.5%) had two or more metastatic sites. Liver, brain, bone, and pleural metastasis were present in 11.6%, 19.9%, 27.1%, and 25.6% of patients, respectively. Median follow-up was 10.8 months (IQR 4.5–16.6).

### 3.2. Early Progressors

A total of 321 out of 336 patients had complete information on disease progression and were therefore considered for early progression evaluation. EP occurred in 137 out of 321 patients (42.7%). At univariate analysis, female sex, ECOG PS 1 or 2, concomitant steroid treatment, high tumor burden, more than two metastatic sites, and the presence of liver, bone, and pleural metastasis, were associated with a significantly higher risk of developing fast progression. Among these, six features were confirmed as independent prognostic factors in multivariate analysis: female sex, ECOG PS 1 or 2, concomitant steroid treatment, more than two metastatic sites, the presence of liver and pleural metastasis (Table 2).

### 3.3. Development of EP Prognostic Score Model

By combining the six factors that remained significant at the multivariate analyses in the logistic model with PFS < 3 months as the endpoint, we created a prognostic score based on regression β coefficients as follows: patients were given zero points for each of ECOG PS of 0, male gender, only one metastatic site, no use of corticosteroids and neither pleural nor liver metastases (reference category for each item); one point for ECOG PS of 1; two points for each of presence of two or more metastatic sites, female gender, use of corticosteroids, presence of pleural metastases, presence of liver metastases; three points for ECOG PS of 2.

The aim of the score was the prediction of a PFS of less than three months. As a result of this process, the score ranged from 0 (best prognosis) to 13 (worst prognosis). The area under the curve (AUC) of the model was 0.76 (95% CI: 0.70–0.81). We then used a three-risk group stratification as follows: score 0–2 (good prognosis), score 3–6 (intermediate prognosis), score 7–13 (poor prognosis). 

According to the score stratification, the probability of experiencing PFS < 3 months is shown in Table 3.

### 3.4. Survival Outcomes

In the overall study population, the median PFS was 3.8 months (95% CI: 2.9–4.7). The median OS was not reached. The 2-year survival rate was 53.3% in the overall population.

**Figure 1:**
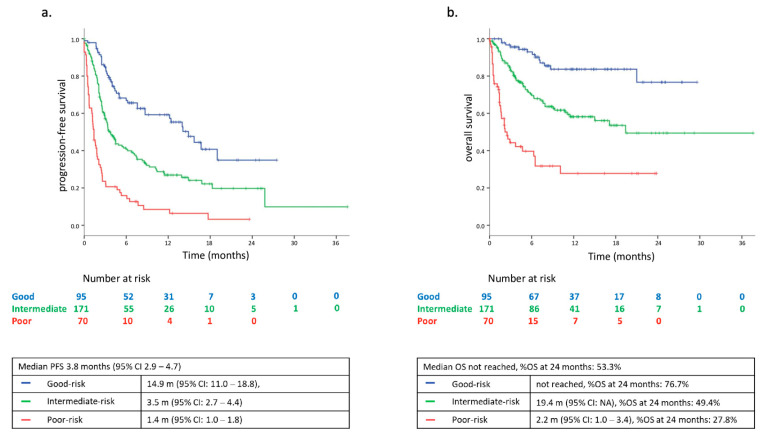
(**a**) Progression free survival (PFS) according to the three risk-groups of the FP prognostic-score model in the study population (N = 336); (**b**) overall survival (OS) according to the three risk-groups of the FP prognostic-score model in the study population (*n* = 336).

We then evaluated the survival outcomes according to the three risk categories of our prognostic score model. Patients in the good-risk group had a median PFS of 14.9 months (95% CI: 11.0–18.8), whereas in the intermediate-risk and the poor-risk groups, the median PFS was 3.5 (95% CI: 2.7–4.4) and 1.4 months (95% CI: 1.0–1.8), respectively (Figure 1a). The hazard ratio (HR) for PFS was significantly higher in intermediate- compared to good-risk (HR 2.13; 95% CI: 1.50–3.04) and in poor-risk compared to good-risk group (HR 5.46; 95% CI: 3.67–8.10).

Consistently, the median OS was not reached in the good-risk group; it was 19.4 months (95% CI: NA) in the intermediate-risk group, and 2.2 months (95% CI: 1.0–3.4) in the poor-risk patients. The 2 year survival rates were 76.7, 49.4, and 27.8% in the three risk categories, respectively (Figure 1b; Supplementary Figure S1; Supplementary Table S1). HR for OS was significantly higher in intermediate- and poor-risk versus good-risk group (HR 3.13; 95% CI: 1.72–5.71, and HR 9.27; 95% CI: 4.93–17.41, respectively).

## 4. Discussion

We examined different clinical factors associated with fast progression in metastatic NSCLC patients treated with first-line pembrolizumab monotherapy. We observed early progression in 42.7% of evaluable patients. This finding is in line with previous reports on the detrimental effects of mono-immunotherapy and confirms the unmet need of identifying prognostic and predictive biomarkers and clinical features to guide better patients’ selection. 

In our retrospective study, we identified six independent prognostic factors associated with the risk of developing EP: sex, ECOG PS, steroid use, number of metastatic sites, liver and pleural metastasis. Female patients, despite the confirmed overall OS benefit over standard chemotherapy, have been shown to derive lower benefit compared to males across the main clinical trials of mono-immunotherapy, both in first-line and further line setting [15]. Our findings confirm a potential negative prognostic impact of the female sex in patients receiving pembrolizumab monotherapy as front-line treatment.

ECOG PS is a well-recognized prognostic factor for lung cancer patients. In particular, ECOG PS 2 patients represent an unfavorable category, characterized by reduced survival outcomes compared to patients with PS 0 or 1 [15,16,17]. Of note, only 13.1% of patients in our study population presented with an ECOG PS ≥ 2, reflecting a known poor prognosis category. However, the higher EP-risk was consistent in PS 1 compared to PS 0 patients, as well. 

Moving forward, the use of concomitant corticosteroids (≥10 mg of prednisone equivalent) was associated with lower performance and reduced OS in patients with NSCLC treated with immune checkpoint blockade [15,18]. Despite the wide use of corticosteroids in the lung cancer setting, their immune-suppressive role as factors impairing ICI activity is highly debated. According to different retrospective study results, the negative prognostic role of concomitant corticosteroids appears limited to patients receiving those for palliation and not confirmed in those receiving corticosteroids for cancer-unrelated conditions, including the treatment of immune-related adverse events [19,20]. In our study, 35.1% of patients received baseline concomitant steroids, and this subgroup of patients had a higher risk of experiencing PFS < 3 months. Due to the multicenter retrospective nature of this work, the reason for baseline steroid assumption was not available for all the included patients, and therefore it was not analyzed as a stratification factor.

Regarding liver metastasis, they are associated with poor prognosis in lung cancer and with immune suppressive microenvironment [21,22]. Liver metastasis confirmed to be predictive of worse clinical outcomes across the pivotal mono-immunotherapy trials in NSCLC [15]. Our data, consistently with these pieces of evidence, provide additional knowledge specifically related to the higher risk of EP in the first-line setting with pembrolizumab in patients with liver metastasis. 

The role of metastatic pleural involvement is crucial because pleural metastases are not easy to assess and often not deeply investigated as a prognostic factor. In particular, in the absence of confirmed pleural nodes, the presence of pleural effusion appears challenging to discriminate between a pathologic and a reactive phenomenon, without a cytological evaluation. However, growing pieces of evidence are emerging about a negative prognostic role of metastatic pleural site. Recently, pleural effusion was an independent negative prognostic factor in NSCLC patients receiving ICI or ICI combinations in different treatment lines, with one-third of patients with pleural effusion experiencing early death [8]. 

Of note, the number of metastatic sites was an independent prognostic factor. In contrast, the high tumor burden was not, suggesting that the multiorgan extension of the disease and not its volume alone has an impact on patients’ outcomes.

Major limitations in our study are the retrospective nature, with data from clinical records across different institutions, and the lack of a control group. In particular, in the absence of comparison with first-line chemotherapy or chemo-immunotherapy combinations, the prognostic value of the considered clinical factors in those receiving pembrolizumab monotherapy cannot be translated into a predictive evaluation of treatment response. To this concern, the treatment setting of front-line pembrolizumab in our country (NSCLC with PD-L1 ≥ 50%) does not include alternative treatment approaches according to regulatory approvals and, therefore, makes it impossible to settle on a proper control cohort to investigate the predictive value of identified prognostic factors.

Another limitation is that only clinical factors were considered in our model, whereas no biomarker evaluation was included. In particular, within the high PD-L1 range, it could be useful to assess the value of PD-L1 as a prognostic marker together with clinical features based on recent evidence [23]. Moreover, recent retrospective studies identified the negative prognostic role of high neutrophil to lymphocyte ratio (NLR, with different cut-offs), as well as albumin and lactate dehydrogenase in blood, and included it into prognostic models in addition to clinical features including ECOG PS, steroid use, metastatic sites in patients receiving ICIs [8,24,25]. However, biomarker assessment was not included in our study, as it was designed to specifically evaluate clinical features that are routinely used in clinical practice for patient evaluation and treatment selection.

Our proposed prognostic model is based on retrospective data and needs prospective validation. Potentially, it could be a helpful tool to identify, in the poor-risk patient group, almost 80% probability to develop early progression (median PFS 1.4 months) and shorter OS (median OS 2.2 months) in the first-line setting. Therefore, we are planning a prospective study to validate this clinically based prognostic scoring system and to investigate additional biomarker evaluation, including NLR and continuous PD-L1 values. 

## 5. Conclusions

In conclusion, we were able to identify six independent prognostic factors in high PD-L1 NSCLC patients receiving the first-line pembrolizumab associated with early progression. We built a prognostic score model based on these clinical factors that have easy applicability in clinical practice and might provide a helpful tool to better select patients for first-line treatment in high PD-FL1 NSCLC. In particular, the indication of ICI monotherapy should be carefully considered in poor-risk patients, who might deserve earlier radiologic assessment after ICI initiation to detect in advance the occurrence of detrimental effects, and eventually define early treatment switch.

## Figures and Tables

**Table 1:** Clinical characteristics of the study population.

Clinical Characteristics	Overall Population (*N* = 336)
Age, median (range)	69 (36–86)
Sex, *n* (%)	
M	227 (67.6%)
F	109 (32.4%)
Smoking status, *n* (%)	
Ever	285 (84.8%)
Never	46 (13.7%)
unknown	5 (1.5%)
ECOG PS, *n* (%)	
0	121 (36%)
1	171 (50.9%)
≥2	44 (13.1%)
Histology, *n* (%)	
Squamous	62 (18.5%)
Non-squamous	264 (78.6%)
NOS	10 (2.9%)
Comorbidities, *n* (%)	
Yes	245 (73%)
No	90 (26.8%)
unknown	1 (0.2%)
Concomitant steroids, *n* (%)	
Yes	118 (35.1%)
No	218 (64.9%)
Metastatic sites, *n* (%)	
0	1 (0.3%)
1	51 (15.2%)
≥2	284 (84.5%)
Liver metastases, *n* (%)	
Yes	39 (11.6%)
No	297 (88.4%)
Pleural metastases, *n* (%)	
Yes	86 (25.6%)
No	250 (74.4%)
Brain metastases, *n* (%)	
Yes	67 (19.9%)
No	269 (80.1%)
Bone metastases, *n* (%)	
Yes	91 (27.1%)
No	245 (72.9%)

**Table 2:** Univariate and multivariate logistic regression for the association of clinical characteristics with early progression.

Clinical Characteristics	Univariate OR (95% CI)	Multivariate OR (95% CI)
Sex (M vs. F)	0.59 (0.37–0.94) *p* = 0.03	0.49 (0.29–0.84) *p* = 0.01
Age (≥68 vs. <68)	0.82 (0.53–1.26) *p* = 0.36	
Smoking habit		
- light/past vs. never	0.64 (0.34–1.22) *p* = 0.18	
- current vs. never	0.74 (0.37–1.46) *p* = 0.38	
Comobidity (yes vs. no)	0.85 (0.52–1.38) *p* = 0.51	
ECOG PS		
- 1 vs. 0	2.14 (1.29–3.57) *p* = 0.003	1.77 (0.99–3.18) *p* = 0.05
- 2 vs. 0	5.89 (2.79–12.44) *p* < 0.0001	5.60 (2.46–12.71) *p* < 0.0001
Concomitant steroids (yes vs. no)	2.46 (1.55–3.89) *p* < 0.0001	2.28 (1.37–3.79) *p* = 0.001
Concomitant antibiotics (yes vs. no)	0.89 (0.51–1.55) *p* = 0.68	
High tumor. Burden (yes vs. no)	2.13 (1.33–3.43) *p* = 0.002	
Number of metastatic sites (≥2 vs. ≤1)	3.08 (1.95–4.86) *p* < 0.0001	2.31 (1.43–3.72) *p* = 0.001
Brain metastasis (yes vs. no)	1.38 (0.81–2.37) *p* = 0.23	
Liver metastasis (yes vs. no)	2.51 (1.32–4.79) *p* = 0.005	2.27 (1.07–4.86) *p* = 0.03
Bone metastasis (yes vs. no)	2.39 (1.48–3.840) *p* < 0.0001	
Pleural involvement (yes vs. no)	2.01 (1.23–3.26) *p* = 0.005	2.18 (1.29–3.69) *p* = 0.004
Lung metastasis (yes vs. no)	1.41 (0.89–2.22) *p* = 0.14	
Adrenal metastasis (yes vs. no)	1.55 (0.88–2.73) *p* = 0.13	
Lymph node metastasis (yes vs. no)	0.80 (0.47–1.34) *p* = 0.40	

**Table 3:** Proposed prognostic score model for early progression in patients receiving 1L mono-immunotherapy.

Score (Points) ^1^	PFS ≥ 3 Months (Number of Patients/%)	PFS < 3 Months (Number of Patients/%)
Score 0–2	76 (84.4%)	14 (15.6%)
Score 3–6	92 (56.8%)	70 (43.2%)
Score 7–13	16 (23.2%)	53 (76.8%)
Total	184 (57.3%)	137 (42.7%)

^1^ Scoring assignment: 0 point: ECOG PS 0, male gender ≤1 metastatic site, no corticosteroids, neither pleural nor liver metastases; 1 point: ECOG PS 1; 2 points: ≥2 metastatic sites, female gender, use of corticosteroids, pleural metastases, liver metastases; 3 points: ECOG PS 2.

## Supplementary Materials

The following are available online at https://www.mdpi.com/article/10.3390/cancers13122935/s1, Figure S1: Receiver Operating Characteristic (ROC) curve of the model developed; Table S1: Beta coefficients used to build the score system.
