# Supplementary material for: Early Progression in Non-Small Cell Lung Cancer (NSCLC) with High PD-L1 Treated with Pembrolizumab in First-Line Setting: A Prognostic Scoring System Based on Clinical Features
Source: Cancers (Basel). 2021 Jun 11;13(12):2935. doi: 10.3390/cancers13122935 (PMC8230881; doi:10.3390/cancers13122935)
Supplement: Supplementary file 1 [file cancers-13-02935-s001.zip › cancers-1230445-supplementary.pdf]

Figure S1: Receiver Operating Characteristic (ROC) curve of the model developed

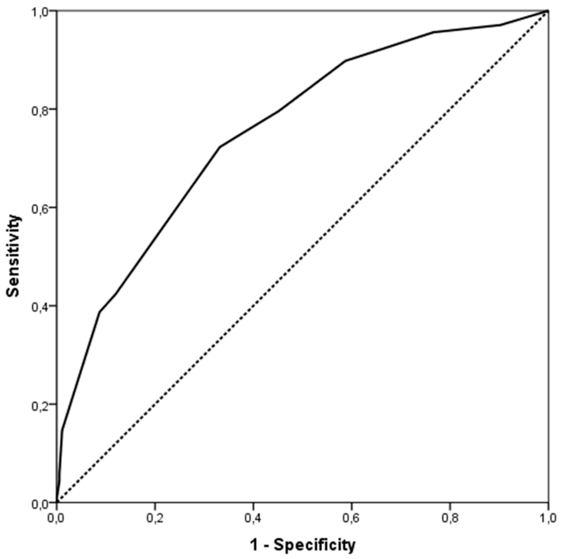

The curve shows the predictive power of the scoring system for early progression (PFS < 3 months).

The AUC of the model was 0.76 (95% CI: 0.70-0.81).

Table S1. Beta-coefficients used to build the score system.

Clinical characteristics	beta coefficient (S.E.)
SEX (F vs. M)	0.71 (0.28)
ECOG PS	
(1 vs. 0)	0.57 (0.30)
(2 vs. 0)	1.72 (0.42)
CONCOMITANT STEROIDS (yes vs. no)	0.82 (0.26)
NUMBER OF METASTATIC SITES (≥2 vs. ≤1)	0.78 (0.27)
LIVER METS (yes vs. no)	0.82 (0.39)
PLEURAL METS (yes vs. no)	0.79 (0.29)

S.E. standard error
